# Extreme hypernatremia after a laparoscopic hysterectomy and bilateral salpingo-oophorectomy: a case report and literature review

**DOI:** 10.3389/fsurg.2024.1462525

**Published:** 2024-10-15

**Authors:** Fei Ding, Xin Nie, Yuemei Chen, Minjin Wang, Yong He

**Affiliations:** Department of Laboratory Medicine, West China Hospital, Sichuan University, Chengdu, China

**Keywords:** nephrogenic diabetes insipidus (NDI), *AVPR2*, missense mutation, water deprivation and vasopressin test, X-linked recessive

## Abstract

Congenital nephrogenic diabetes insipidus (NDI) primarily arises from an X-linked recessive inheritance caused by mutations in the *AVPR2* gene, which is responsible for approximately 90% of cases. This condition has an incidence rate of 4–8 per million male live births, with females being much less frequently affected. Symptoms typically manifest shortly after birth, predominantly in males. The key clinical features of NDI include excessive urination (polyuria), compensatory excessive thirst (polydipsia), cognitive impairment, consistently low urine specific gravity, dehydration, and imbalances in electrolyte levels. This case study highlights an unusual occurrence of NDI in a 50-year-old Chinese woman attributed to a mutation in the *AVPR2* gene. For more than a year, she had been suffering from excessive urination and severe thirst. The patient, who had undergone surgery for cervical cancer, developed polyuria and hypernatremia postoperatively. Initial laboratory analyses revealed normal blood sodium and chloride levels but reduced urine osmolality and specific gravity. Imaging assessments revealed no irregularities. To validate the diagnosis of NDI, she participated in a water deprivation and vasopressin test. Subsequent genetic tests revealed a thymine (T) to adenine (A) mutation, leading to a missense mutation in the *AVPR2* gene. As part of her treatment, she was placed on a low-sodium diet and prescribed oral hydrochlorothiazide and indomethacin for 1 month, resulting in a marked improvement in her symptoms. To the best of our knowledge, this is the first documented case of NDI diagnosed postoperatively in an older female patient with *AVPR2* heterozygosity. This case highlights an unusual instance of an X-linked recessive clinical presentation of NDI in an elderly female patient. This study also underscores the importance of conducting water deprivation, vasopressin tests, and genetic testing in establishing the underlying cause for individuals diagnosed with NDI.

## Introduction

Diabetes insipidus (DI) is considered a rare condition, affecting approximately 1 in 25,000 individuals. The most prevalent type is central diabetes insipidus (CDI), which stems from either an acquired or genetic issue in the neurohypophysis, resulting in a decrease in arginine vasopressin (AVP) production and release. Among the different types, acquired cases far outnumber those inherited genetically (1). To identify potential pituitary abnormalities that might contribute to diabetes insipidus, magnetic resonance imaging (MRI) of the pituitary gland can be conducted if needed. In cases of congenital nephrogenic DI, approximately 90% are linked to an X-linked recessive mutation in the *AVPR2* gene, with an incidence rate of 4–8 per million male births (1). This genetic mutation leads to the renal tubules being unresponsive to AVP. Symptoms typically surface shortly after birth, predominantly affecting males, who often experience growth delays, whereas females are rarely affected (2). Following the administration of vasopressin, there was no reduction in urine volume or increase in urine specific gravity, clearly distinguishing this condition from CDI (1).

When the serum osmolality increases, the hypothalamus activates signals from the supraoptic and paraventricular nuclei, which travel through magnocellular neurons to prompt the posterior pituitary to release AVP (3). This vasopressin then travels to the distal convoluted tubules (DCTs) in the kidneys, where it binds to specific receptors (3). This interaction triggers the movement of aquaporin-2 channels from the cytoplasm to the apical membrane of the DCT, facilitating the reabsorption of water back into the bloodstream (3). Consequently, the osmoreceptors within the hypothalamus sense a decrease in serum osmolality and subsequently decrease AVP production (3).

The gold-standard test for diagnosing DI involves restricting fluid intake while closely observing urinary output, urine osmolality, plasma sodium levels, and plasma osmolality. Following the administration of desmopressin (DDAVP), the patient's urine osmolality is assessed and juxtaposed with the levels recorded prior to DDAVP (4). Typically, at the conclusion of the test, healthy participants should exhibit a urine osmolality exceeding 800 mOsm/kg, with no significant change post-DDAVP. In contrast, nephrogenic diabetes insipidus (NDI) and CDI patients usually have a urine osmolality less than 300 mOsm/kg (3). The reaction to DDAVP serves to distinguish between NDI and CDI (3); patients with CDI often see an increase in urine osmolality greater than 50%, whereas those with NDI experience an increase of less than 50% (3).

NDI is an X-linked rarely seen disorder that typically does not manifest in middle-aged women. We documented an unusual instance of late-onset congenital NDI in an elderly female patient, who presented with severe hypernatremia following laparoscopic hysterectomy and bilateral salpingo-oophorectomy. Ultimately, she was diagnosed with congenital NDI through water deprivation and vasopressin testing, as well as single whole-exome sequencing [next-generation sequencing (NGS)] conducted in the laboratory. Following this diagnosis, she and her son received appropriate treatment and successfully resumed their normal lives.

## Patient information

A 50-year-old woman, who had a history of obesity and cervical cancer, visited the Endocrinology Department after undergoing laparoscopic surgery. She expressed concerns about experiencing excessive thirst and urination for over a year. The patient reported consuming approximately 10–12 L of water daily and noted that her urine output matched that amount. She had to drink water every 3 h, even during the night. If she missed a drink, she would have a dry mouth, become tearful, and feel weak, but these symptoms would improve after rehydration.

Previous history: Overall, the patient was in a stable condition with no reported infections. She had previously undergone treatment for cervical cancer, which included a laparoscopic hysterectomy and bilateral salpingo-oophorectomy, and had a history of dialysis. The surgery proceeded without complications, and there was no need for a blood transfusion. Recovery after the operation was smooth. Pathology revealed a medium to poorly differentiated squamous cell carcinoma infiltrating the superficial muscle layer in the cervix, but no malignant cells were detected in the surrounding tissues. In the left pelvic lymph nodes, there were 13 instances of reactive hyperplasia, whereas the right side contained 20 such instances. The International Federation of Gynecology and Obstetrics stage was determined to be IB1.

Personal history: Professional farmer, never been to pastoral areas, no history of prostitution, and a clean slate free from any background in prostitution, substance abuse, smoking, or drinking.

Menstrual history: Menarche occurred at 14 years, with a menstrual period lasting 4 days and a cycle duration of 32 days. Her most recent menstrual cycle occurred in March 2023, after which she underwent a laparoscopic hysterectomy along with a bilateral salpingo-oophorectomy within the same month.

Marriage and childbearing history: She married at 20 and was fortunate to have a healthy partner. However, the marriage did not last and she chose not to remarry afterward, and there is no history of widowhood. Together, they had one son, who is now 28 and in good health. Throughout her life, she experienced four pregnancies, resulting in one natural birth and three abortions.

Family history: Her father passed away due to a cerebral hemorrhage, while her mother died under circumstances that remain unclear. She has two older brothers, three older sisters, and one younger brother, all of whom are in good health. There was no reported family history of any medical conditions or genetic issues.

The physical examination results were as follows: 24-h intake, 6,200 ml; 24-h urine volume, 6,800 ml; temperature, 36.4℃; heart rate, 80 times/min; blood pressure, 124/74 mmHg; respiration rate, 19 breaths/min; height, 156 cm; weight, 71 kg; BMI, 29.17 kg/m^2^; waist, 90 cm; hip, 103 cm; and waist‒hip ratio, 0.87. The examinations of the head, neck, chest, abdomen, limbs, and spine were not unique.

Seven months previously, the patient underwent a laparoscopic hysterectomy along with a bilateral salpingo-oophorectomy at another facility due to cervical cancer. Following surgery, she experienced polyuria and hypernatremia. After undergoing a session of continuous renal replacement therapy, her hypernatremia improved. However, upon discharge, she continued to suffer from intense thirst and frequent urination. The patient expressed that her severe hypernatremia stemmed from a lengthy period of water restriction before and after her cervical cancer operation, which put her life at risk. Consequently, she sought additional treatment at our hospital.

The patient presented with excessive thirst and frequent urination, consuming between 10 and 12 L of water daily, with urine output mirroring intake. This led to a preliminary diagnosis of DI. Considering her symptoms and age, it is essential to differentiate her condition from the following conditions: (1) psychic polydipsia, which is primarily observed in middle-aged women and is often associated with anxiety, insomnia, and a dry mouth that subsequently develops into increased thirst and urination. However, establishing this diagnosis requires other underlying disorders being ruled out. (2) Hypercalcemia, which frequently results in polydipsia or polyuria and is typically observed in patients with primary hyperparathyroidism, vitamin D toxicity, multiple myeloma, or cancer-related bone metastases. In this case, the patient had a history of cervical cancer and surgical intervention but presented no signs of bone pain or anemia, with no other related findings. (3) Patients with Sjögren's syndrome generally exhibit dry mouth without accompanying dry eyes, joint pain, or any other symptoms suggestive of immune system disorders, necessitating further checks of immune markers for accurate differentiation. (4) Chronic kidney disease, particularly renal tubular disorders, along with conditions such as hypokalemia and hypercalcemia, can impair the ability of the kidneys to concentrate urine, leading to symptoms such as increased urination and thirst. However, the clinical signs are more reflective of the underlying condition, and excessive urination tends to be milder. (5) In patients with diabetes, elevated blood glucose levels can trigger osmotic diuresis, resulting in an increased urine output. However, in this scenario, the patient lacked a previous diagnosis of diabetes, which undermines this potential diagnosis. (6) If hyperthyroidism prompts an increase in bone calcium metabolism and a urinary excretion of calcium during its initial stages, it may lead to temporary polyuria. Nonetheless, the patient did not exhibit symptoms such as agitation, excessive sweating, or weight loss. Thyroid function can be thoroughly evaluated by appropriate testing.

The laboratory findings indicated that the urine osmolality was notably low at 54 mOsm/kg H_2_O (normal range, 600–1,000 mOsm/kg H_2_O), whereas the urine specific gravity was 1.002 (normal range, 1.003–1.030). The plasma osmolality was measured at 299 mOsm/kg H_2_O (normal range, 275–303 mOsm/kg H_2_O). Sodium levels were recorded at 143.7 mmol/L (normal range, 135–145 mmol/L), and potassium levels slightly decreased at 3.35 mmol/L (normal range, 3.50–5.30 mmol/L), as outlined in Table 1. Imaging revealed no abnormalities in the heart, gallbladder, pancreas, spleen, kidneys, ureters, bladder, adnexa, or pelvic cavity. However, liver ultrasound indicated the presence of fatty liver. A brain MRI returned normal results.

**Table 1:** Laboratory results (all specimens are serum, unless indicated).

Test	Result	Reference interval
Osmotic pressure, urine	54 mOsm/kg H_2_O	600–1,000
Specific gravity, urine	1.002	1.003–1.030
Osmotic pressure, plasma	299 mOsm/kg H_2_O	285–295
Thyroid-stimulating hormone	2.61 mIU/L	0.27–4.20
Thyroxine, free	18.7 pmol/L	12.0–22.0
Aspartate transaminase	16 U/L	<40
Alanine transaminase	16 U/L	<35
Alkaline phosphatase	69 U/L	50–135
Bilirubin	12.9 μmol/L	5.0–28.0
Blood urea nitrogen	1.6 mmol/L	2.6–7.5
Creatinine	46 μmol/L	48–79
Glucose	5.11 mmol/L	3.90–5.90
Bicarbonate	22.3 mmol/L	18.0–28.0
Sodium	143.7 mmol/L	135–145
Potassium	3.35 mmol/L	3.50–5.30
Chloride	107.4 mmol/L	99–110
Calcium	2.31 mmol/L	2.11–2.52
Albumin	42.9 g/L	40.0–55.0
Total protein	66.3 g/L	65.0–85.0
Alpha fetoprotein	2.89 ng/ml	<7
Carcinoembryonic antigen	1.11 ng/ml	<5
Serum carbohydrate antigen 153 (CA-153)	5.52 U/ml	<24
CA-199	21.80 U/ml	<30
CA-125	10.10 U/ml	<25
CA72-4	2.11 U/ml	<6.5
Cytokeratin 19 fragment	1.33 ng/ml	<3
Neuron-specific enolase	21.8 ng/ml	<20.4

The patient's serum osmolality was recorded at more than 295 mOsm/kg, whereas her urine osmolality decreased below 200 mOsm/kg, and the urinary specific gravity was measured at less than 1.003. Following her cervical cancer surgery, she developed hypernatremia due to extended water deprivation, which escalated to the point at which dialysis became necessary. Some individuals are diagnosed with NDI after experiencing postoperative polyuria or hypernatremia (5). On the basis of these findings, a diagnostic strategy was devised that included administering a water deprivation test to affirm the diagnosis of DI (Table 2), with further functional assessments to follow if needed. A subcutaneous injection of 2 µg of DDAVP was also given to ascertain the specific type of DI (Table 3). Post-DDAVP treatment, urine osmolality decreased to less than 300 mOsm/kg, and in the NDI group, it increased by less than 50%. Consequently, the diagnosis of NDI was unequivocally established.

**Table 2:** The water deprivation test.

Time	Urine volume (ml)	Urine specific gravity	Urine osmotic pressure (mOsm/kg H_2_O)	Plasma osmotic pressure (mOsm/kg H_2_O)	Serum sodium (mmol/L)
22:00	400	1.001	89	318	142.0
First urination at night	900	1.003	85	—	—
Second urination at night	900	1.002	84	—	—
6:00	500	1.002	87	327	148.5
7:00	400	1.003	92	332	153.6

**Table 3:** The water deprivation and vasopressin test.

Time	Urine volume (ml)	Urine specific gravity	Urine osmotic pressure (mOsm/kg H_2_O)	Plasma osmotic pressure (mOsm/kg H_2_O)	Serum sodium (mmol/L)
6:00	400	1.001	44	310	141
7:00	300	1.002	65	—	—
8:00	510	1.001	53	312	145.9
9:00	400	1.002	59	—	—
10:00	200	1.002	71	319	149.5
11:00	350	1.002	71	—	—
12:00	320	1.002	77	326	153.9
13:00 (desmopressin 2 μg)	200	1.002	84	332	154.9
14:00 (1 h after arginine vasopressin)	400	1.002	81	326	154.3
15:00 (2 h after arginine vasopressin)	200	1.002	98	331	155.9

Upon further inquiries into her family background, she revealed that she was the sixth offspring of a non-consanguineous marriage. She asserted that none of her six siblings or their children exhibited any symptoms. However, she did mention that her 28-year-old son, who is single, had been experiencing polydipsia and polyuria since he was a child, although the precise course of these symptoms remains unclear. NGS was conducted at the Center for Precision Medicine, West China Hospital of Sichuan University, and revealed a heterozygous mutation in the *AVPR2* gene. A heterozygous mutation at nucleotide 971, where T switches to A (c.971T>A), results in a missense mutation that changes amino acid 324 from isoleucine to asparagine (p.Ile324Asn). According to the American College of Medical Genetics and Genomics (ACMG) guidelines, this mutation has been tentatively classified as likely pathogenic with the following supporting criteria: PM1 indicates that it resides in a mutation hotspot; PM2 indicates a frequency of zero in the normal population database; PM5 emphasizes that a known pathogenic variant resulting in a different amino acid at the same codon has been documented (specifically, c.972C>G, p.Leu324Met, which is absent from ClinVar but is noted in the disease-associated mutation within the Human Gene Mutation Database, HGMD); and PP3, when using the comprehensive prediction tool REVEL, indicates a potentially harmful effect, with other programs such as SIFT, PolyPhen_2, MutationTaster, and GERP + all labeling the result as harmful. There is no documented correlation for this site in the literature, nor could any pathogenicity analysis for it be located in the ClinVar database. In addition, owing to the lack of parental samples, the origin of the genetic variation remains unconfirmed.

To date, diagnosing congenital NDI has been straightforward for this woman. The patient was advised to follow a low-sodium diet and was also prescribed 25 mg of oral hydrochlorothiazide once a day over the course of a month. This regimen resulted in marked improvements in her clinical condition. Consequently, her symptoms improved considerably, as reflected by her daily urine output, ranging from 3,000 to 4,000 ml.

## Discussion

This case describes a unique occurrence of an X-linked recessive clinical manifestation known as NDI in an older female. The significance of conducting water deprivation and vasopressin tests, along with genetic testing, for accurately diagnosing the underlying causes of NDI in patients is also highlighted.

Our research represents the first account of an elderly woman diagnosed with an X-linked recessive condition following surgery, characterized by NDI and confirmed through NGS, revealing a heterozygous status—an unusual finding in females. However, a limitation we face is the absence of pedigree results, and her son declined to participate in testing. Another limitation is that her laparoscopic procedure and subsequent dialysis took place at another hospital. Consequently, our understanding of her health status during that period was confined to medical documentation, examination reports, and patient accounts gathered from other hospitals.

**Figure 1:**
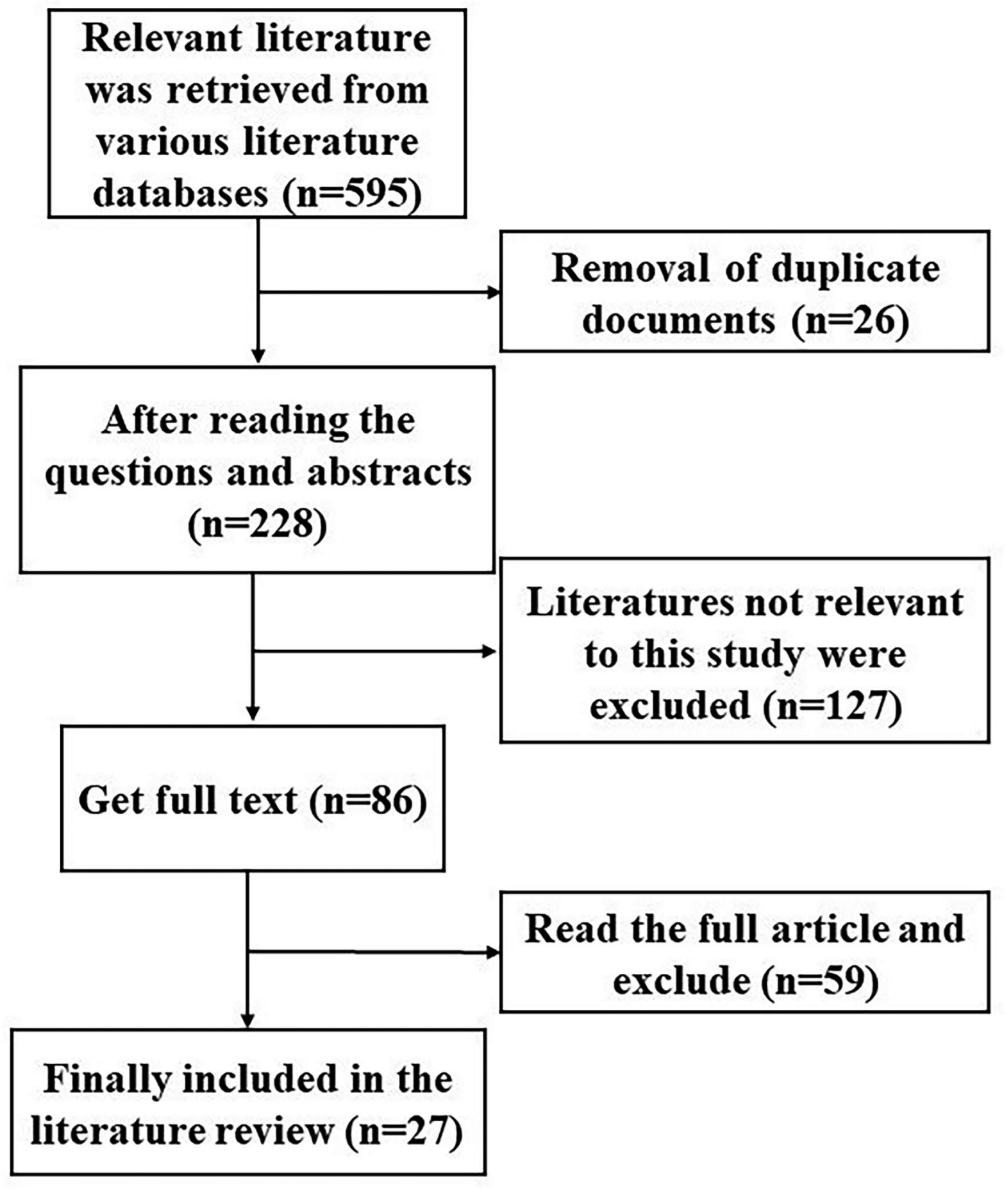
Flow chart for the selection of papers to be included in the review.

Our paper is a narrative review that conforms to the Preferred Reporting Items for Systematic reviews and Meta-Analyses (PRISMA) criteria. The first searches of the databases revealed a total of 595 papers; according to the PRISMA checklist and inclusion criteria, a more accurate identification was performed, with 27 papers selected for literature review (Figure 1). Following water deprivation and vasopressin testing, the diagnosis of NDI in the patient became evident. Genetic testing subsequently confirmed her congenital delayed NDI. To our knowledge, this is the first postoperative identification of NDI in an elderly female patient with *AVPR2* heterozygotes. The *AVPR2* gene can be found in the Xq28 region of the chromosome, and it is inherited in an X-linked recessive manner (6). As a result, the majority of cases are typically present in males, although heterozygous females exhibit variable expressivity (7). A study conducted in Japan revealed that 25% of women displaying clinical symptoms of NDI were confirmed to be heterozygotes (8). Conversely, a Spanish investigation revealed a prevalence of 50%, although the smaller sample size of carriers (12 as opposed to 64 in the Japanese study) may account for this discrepancy (9). This condition has significant ramifications, particularly as antidiuretic hormone (ADH) treatment is ineffective against polydipsia and polyuria, both of which disrupt her daily life, causing her to awaken for bathroom trips up to twice a night. Additional research is essential to better understand the prevalence of symptoms in female *AVPR2* heterozygotes. Consequently, we recommend that heterozygous females receive closer monitoring, as this would facilitate genetic testing for possible undiagnosed carriers. Doing so will not only ensure their own health but also pave the way for effective family counseling (9).

Most female carriers of *AVPR2* heterozygosity remain asymptomatic; however, a small subset experiences varying degrees of polyuria and polydipsia. In contrast to earlier assumptions, symptomatic females may be more common than previously recognized. Research indicates that female carriers with heterozygous missense mutations in *AVPR2* can exhibit symptoms of NDI, potentially due to skewed X chromosome inactivation (XCI) (10). The pattern of XCI may vary across different tissues (11). Moreover, additional elements, such as environmental factors and DNA methylation, might also influence this condition. In addition, instances of discordant XCI escape have been observed in monozygotic twins, which could affect the variability in phenotypic expression among females (12). A study focusing on monozygotic twins exhibiting distinct NDI phenotypes proposed that the emergence of NDI symptoms is more closely linked to the XCI pattern found in urine sediments than to that found in peripheral leukocytes (13). Another review highlighted that X-linked NDI in female *AVPR2* heterozygotes consistently correlates with skewed XCI, underscoring the importance of conducting XCI studies within this population (14).

Postoperative polyuria is a frequent complication that occurs after extensive surgical procedures. Increased sympathetic nervous system activity during surgery leads to the increased secretion of vasopressin and aldosterone. This surge, along with the substantial intravenous fluids administered to patients, encourages fluid retention (15). Following surgery, however, a decrease in vasopressin and aldosterone levels can trigger the elimination of this retained fluid, leading to polyuria. Importantly, this condition may also be linked to DI, a rare disorder that results in excessive urination due to the decreased sensitivity of the kidneys to antidiuretic hormone in the collecting ducts. To prevent dehydration and elevated sodium levels, it becomes essential for patients to consume ample water. We present a case involving a female patient who, due to cervical cancer, underwent surgery and fasting and subsequently experienced postoperative polyuria and hypernatremia, ultimately being diagnosed with nephrogenic DI. As non-nephrologist physicians are often tasked with managing patients with DI for several conditions, it is imperative that they are knowledgeable about fluid management and electrolyte balance during emergency admissions and similar situations. Patients who are unaware of their NDI may struggle more than those without the condition in managing their fluid and electrolyte levels, especially when unrestricted drinking is not an option (5). Hence, it is crucial to obtain a comprehensive medical history prior to surgery. Many patients may harbor misconceptions about health, such as the belief that an increased water intake is always beneficial, and might not realize that their excessive thirst needs to be communicated to their physician, particularly if they lack medical knowledge.

Endometrial cancer (EC) is the most common gynecologic cancer, and 7% of ECs are diagnosed in patients younger than 45 years. A fertility-sparing approach for grade 1 endometrioid adenocarcinoma limited to the endometrium is feasible (16). With respect to pharmaceutical targets, progestins, particularly medroxyprogesterone acetate (MPA) and megestrol acetate (MA), are the most commonly employed agents in the conservative treatment of early-stage EC (17). This new molecular classification provides new strategies for identifying patients at a high or low risk of progression and relapse and improving the selection of patients for a fertility-sparing approach (18). In addition, adequate biomarkers may be valuable prognostic tools, and micro RNAs might be promising tools for patient evaluation (19).

For the diagnosis of malignancies, vitrification can be used as a cryopreservation technique for human oocytes and for the preservation of female fertility (20). Higher pregnancy rates and better perinatal outcomes are associated with frozen embryo transfers than with fresh techniques (21). During pregnancy, along with investigations into the usually increased risk of preeclampsia (22) and postpartum hemorrhage (23), it is also suggested to perform a non-invasive prenatal test (NIPT) (24) at the beginning of pregnancy, follow neonatal outcomes, and carry out a long-term follow-up of children born (25, 26) from frozen embryos to focus on preeclampsia and postpartum hemorrhage treatments (27). During pregnancy, these patients may feel less pain with a waterbirth (28) in labor as well. In these patients, it can be helpful to use contrast agents (29) during pregnancy.

Hypernatremia occurs via two main mechanisms in cancer patients: net water loss or excessive salt intake (30). For example, a recent case study reported that a patient undergoing follow-up for cervical cancer believed that bay salt would cure cancer. The patient had been taking four teaspoons of bay salt a day, leading to extremely severe hypernatremia (31). We reported a case of hypernatremia due to prolonged fasting during surgery. The woman was diagnosed with the rare disease NDI.

**Figure 2:**
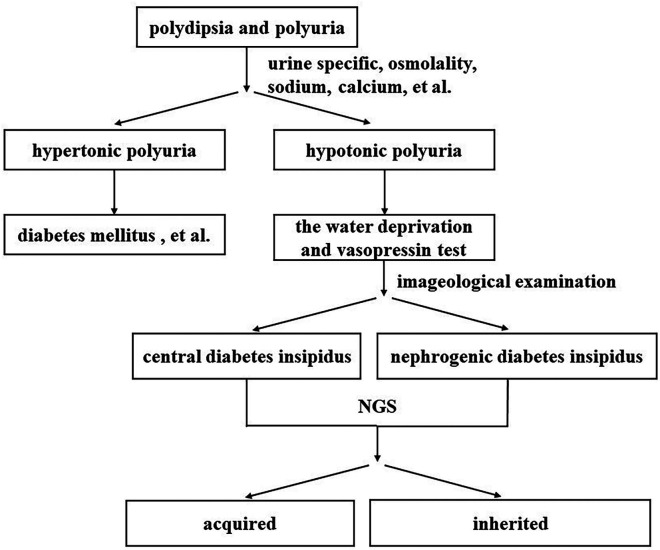
Clinical polydipsia polyuria diagnostic pathway map.

In this case study, we reported a case of rare disease and outlined the diagnostic framework for patients experiencing clinical polydipsia and polyuria (Figure 2). We highlight the importance of conducting diagnostic water deprivation and vasopressin tests for those with these symptoms. In addition, NGS testing proves invaluable in distinguishing between congenital and acquired forms of the disease, particularly in the context of rare disorders.

## Patient perspective

Upon receiving my cervical cancer diagnosis, my world felt like it had been engulfed in shadows, particularly when I considered the prospect of my son facing the future without me. To add to the turmoil, I faced a critical episode of hypernatremia following my surgery, necessitating immediate dialysis. Thankfully, West China Hospital stepped in to diagnose and manage my diabetes insipidus. Gradually, my concerns for my son began to ease. I wish to share my journey with a wider audience and, after my passing, I hope to contribute my body to your hospital for research on this grinding disease.
